# Changes in Metabolite Profiles of Chinese Soy Sauce at Different Time Durations of Fermentation Studied by ^1^H-NMR-Based Metabolomics

**DOI:** 10.3390/metabo14050285

**Published:** 2024-05-15

**Authors:** Jalal Uddin, Samra Yasmin, Ghulam Mustafa Kamal, Mufarreh Asmari, Muhammad Saqib, Heyu Chen

**Affiliations:** 1 Department of Pharmaceutical Chemistry, College of Pharmacy, King Khalid University, Abha 61441, Saudi Arabia; 2 Institute of Chemistry, Khwaja Fareed University of Engineering & Information Technology, Rahim Yar Khan 64200, Pakistan; 3 Innovation Academy of Precision Measurement Science & Technology, University of Chinese Academy of Sciences Beijing, Wuhan 430071, China; 4 College of Mechanical and Electronic Engineering, Northwest A&F University, Xianyang 712100, China

**Keywords:** soy sauce, fermentation, metabolites profiling, metabolomics, NMR spectroscopy

## Abstract

Fermentation parameters, especially the duration, are important in imparting a peculiar taste and flavor to soy sauce. The main purpose of this research was to monitor metabolic changes occurring during the various time intervals of the fermentation process. NMR-based metabolomics was used to monitor the compositional changes in soy sauce during fermentation. The ^1^H-NMR spectra of the soy sauce samples taken from the fermentation tanks at 0 to 8 months were analyzed using ^1^H-NMR spectroscopy, and the obtained spectra were analyzed by multivariate statistical analysis. The Principal Component Analysis (PCA) and Partial Least Square Discriminate analysis (PLSDA) revealed the separation of samples fermented for various time durations under identical conditions. Key metabolites shown by corresponding loading plots exhibited variations in amino acids (lysine, threonine, isoleucine, etc.), acetate, glucose, fructose, sucrose, ethanol, glycerol, and others. The levels of ethanol in soy sauce increased with longer fermentation durations, which can be influenced by both natural fermentation and the intentional addition of ethanol as a preservative. The study shows that the variation in metabolite can be very efficiently monitored using ^1^H-NMR-based metabolomics, thus suggestion to optimize the time duration to get the soy sauce product with the desired taste and flavor.

## 1. Introduction

Soy sauce, a versatile condiment with a rich history spanning over 2000 years, is widely used in various cuisines, particularly Japanese, Chinese, and other Asian cuisines, because of its unique aroma and rich flavor [1]. It is made from fermented soya beans, salt, water, and sometimes barley or wheat flour [2]. The sauce-making process involves soaking, cooking, mashing, fermenting and pasteurizing soybeans, wheat flour, koji starter, water, and salt [3]. The fermentation process takes several months, resulting in a flavorful soy sauce with an excellent aroma [4]. Fermentation is a metabolic process that can occur both in the presence and the absence of oxygen, and involves chemical changes in organic substances through the action of enzymes. Soy sauce develops a complex microbial community of fungi, yeast, and bacteria during production. Although soy sauce varies greatly between countries, it shares a two-step fermentation process, namely, koji (solid-state fermentation) and moromi (brine fermentation), in all cases [5,6,7,8]. Humans have used it since ancient times, and it has various applications in producing sour foods, alcoholic beverages, and animal digestion [9]. During fermentation, energy-rich molecules are converted into different organic products [10].

Traditional Chinese soy sauce fermentation is a complex process involving the interaction of microorganisms, biochemical reactions, the development of distinct flavors, moromi fermentation, and pasteurization [11,12]. Fermentation conditions play a crucial role in shaping soy sauce’s microbial development and aroma profile [13]. Factors such as salt concentration, temperature, pH, and fermentation time can influence the composition and activity of microorganisms, thus affecting the final product [14].

The flavoring ingredients in soy sauce enhance food flavor [15], while its coloring agents enhance the visual appeal of dipping or soup mixtures. The adjustment of numerous parameters, including microbial culture medium, temperature, pH, solvent type, fermenting microorganisms, microbial load, and fermentation maturation period, is essential to derive fermented products with the best yield and quality [16]. Significant changes in various chemical components, such as total nitrogen, reducing sugar, water-soluble peptides, and free amino acids, occur during fermentation [17]. Principle biochemical reactions, including protein degradation, sugar hydrolysis, and the Maillard reaction, play essential roles in the formation of flavor compounds in soy sauce [18]. They contribute to the development of complex taste profiles, aromas, and color characteristics that define the unique sensory attributes of soy sauce [19]. The composition of free amino acids (FAAs), reducing sugars, organic acids (i.e., glutamic, malic, citric, acetic, and lactic acids), and salt has been found to correlate with the overall taste of soy sauce; they therefore are of prime importance in flavor formation [20]. Ongoing research continues to explore ways to optimize the processing conditions to derive soy sauce’s best quality and flavor. The optimization requires a balance between flavor development, fermentation time and conditions, microbial interactions, and desired sensory attributes [21].

Among all these factors, fermentation time is one of the most significant factors responsible to the soy sauce’s characteristic aroma and quality [22]. The metabolic profile of soy sauce varies with the variation in fermentation time; thus, the concentration of the metabolites present in the soy sauce also changes during different fermentation periods [14]. The optimal timing for soy sauce fermentation to achieve the best taste and aroma is still debatable. The fermentation time for soy sauce typically ranges from several months to years, and different manufacturers may follow different fermentation durations to achieve their desired product characteristics [23].

Traditionally, soy sauce fermentation can take a few months to over a year, allowing for a gradual and natural flavor development [24]. It was said that the longer the duration, the better the taste and quality of the soy sauce, but very little scientific proof exists for this belief. However, modern production methods have introduced accelerated fermentation processes that aim to achieve similar flavor profiles in a shorter time span [25].

Numerous techniques have been employed to analyze soya sauce products at various periods during fermentation [26]. Currently, metabolomics is entrenched, but it is still an evolving technique that applies to a variety of fields. Various crucial factors and potential biomarkers related to nutrition, taste optimization, product authentication, and several other technological properties of foods are also reported [27].

The collection of metabolites and their interactions in a cell are collectively called the metabolome. Nuclear magnetic resonance (NMR) and mass spectrometry (MS) are the favorites for quantifying the metabolites. NMR spectroscopy generates complex multivariate data. Combining NMR measurements with chemometrics or multivariate statistical analyses can extract valuable information from these intricate spectral datasets through dimensionality reduction [28,29].

Among the preferred methods for pattern recognition analysis, Principal Component Analysis (PCA) and Partial Least square differential analysis (PLS-DA) are widely used, reducing the data dimensionality and allowing focus on the most significant data only. Multivariate data techniques can also identify potential biomarkers among two or more groups or classes [30].

Various chromatographic methods are available and have been used to study the variations in metabolite profiles of soy sauce during the course of fermentation. However, all those are targeted and can focus on a class of compounds at a time. NMR-based metabolomics can be high-throughput and non-targeted techniques revealing changes in all types of metabolites at a time. Therefore, in this study, we used ^1^H-NMR spectroscopy coupled with the chemometrics tools to study the variations in soy sauce’s chemical composition subjected to a prolonged fermentation time of eight months.

## 2. Materials and Methods

### 2.1. Sample Collection

Thirty-seven samples of traditional Chinese soy sauce were taken with courtesy from a Chinese fermented food processing company named Jicheng Soya Sauce Brewing Company, which is located at Suzhou, China. The fermentation broth contained koji, microbes, sugars, organic acids, hydrolytic enzymes, wheat, and soybean proteins, etc. The process employed was liquid fermentation. The manufacturer used a high-salt liquid fermentation medium known as soy sauce koji (Huniang3042). Water, soybean, wheat, and salt were used as ingredients/raw materials. Aerobic fermentation was used in the liquid phase. Fermentation was processed in saline conditions and at a controlled temperature in an 8 by 20–120 cubic meter tank. Soya sauce samples belonged to the same product batch but had different fermentation times ranging from 0 to 8 months. Six samples were taken from 0 months of the brewing process, seven from the second month of brewing, eight from the fourth month of brewing, eight from the sixth month of brewing, and eight from the eighth month of brewing. Samples of various ages were filtered using filter papers and stored in glass bottles in a refrigerator at −20 °C until analysis [31,32].

### 2.2. Sample Preparation

To prepare the samples for ^1^H-NMR spectroscopic analysis, 600 µL was used for each sample. Initially, 60 µL of the sample was dissolved in 480 µL of a 0.05 M buffer solution. Additionally, 60 µL of a 2.5 mM Trimethylsilylpropanoic acid (TSP) solution, prepared in 100% deuterium oxide, was added. The pH of the sample mixture was carefully recorded and maintained at 5.0 ± 0.05 using a phosphate buffer solution (sodium phosphate) with a concentration of 0.05 M. The prepared sample mixtures were kept in clean and labeled vials. Then, one by one, they were subjected to centrifugation for 10 min at 13,000 rpm to separate any particulate matter, and the resulting supernatants were transferred to NMR sample tubes for further analysis. To facilitate chemical shift calibration, TSP was used as an internal standard. All the chemicals used were of analytical grade [33].

### 2.3. ^1^H-NMR Spectroscopic Analysis

The ^1^H-NMR experiments were conducted using a Bruker Avance 600 NMR spectrometer with TXI CryoProbe made by Bruker, Germany. The spectrometer was operated at 600.1699 MHz frequency and 298 K temperature. The ^1^H-NMR spectra were acquired, and Trimethylsilylpropanoic acid (TSP) was used as an internal reference standard with chemical shift at (^1^H, δ 0.00 ppm). For water signal suppression, the presaturation technique was used. ^1^H-NMR spectra were observed with a spectral width of 9000 Hz, an acquisition time of 1.8 s with 1 s repetition time, and a number of data points of 32,000. A total of 128 scans were taken in this analysis [33].

Before Fourier transformation, ^1^H-NMR spectra were apodised at the 0.3 Hz exponential line broadening function, and signals assignment was performed using 2D ^1^H-^1^H TOCSY, HSQC, online spectral databases, the in-house built database, and published data [32].

### 2.4. ^1^H-NMR Data Analysis

Peak shift in ^1^H-NMR spectra poses a significant challenge when conducting multivariate spectral data analysis. Various factors are responsible for this shift, such as instrumental differences, pH, temperature, salt concentration variations, overall dilution factors, and the relative concentration of specific ions. However, the impact of these factors is not uniform across all peaks, making it challenging to extract meaningful information from the spectra. Several steps were taken in the data processing workflow to address this issue [33].

Firstly, auto phase, baseline correction, setting TSP as a reference, and alignment of the peaks were performed using the Mnova (14.3.2) software developed by Mestrelab Research. This step ensured that variations in the overall concentration of compounds in the samples did not bias the analysis. All samples’ data were divided into 0.04 ppm spectral buckets using Mnova prior to exporting the data as an excel file [34].

For the analysis, specific regions of interest were considered, namely, 0.8 to 4.36, 6.80 to 6.92, and 7.16 to 8.72 ppm, while excluding other regions that did not exhibit significant signals, as well as the solvent region. This step ensured that only relevant spectral information was retained for further analysis.

### 2.5. Multivariate Data Analysis

Before conducting the multivariate analysis, the NMR data sets were subjected to bucketing and normalization processes. Bucketing grouped the spectral data into bins or buckets, simplifying the data representation by reducing the number of variables. Normalization ensured that the data were scaled and comparable, considering differences in intensity and concentration. The bucketed and normalized spectral data were then analyzed using MetaboAnalyst (v5.0), a user-friendly interface. After alignment and bucketing, the stacked spectra were normalized based on the total spectral area to compensate for variations in total concentration. This normalization step eliminated the effects of concentration differences on the subsequent analysis [35].

In the analysis, the Pareto scaling method was employed for all multivariate analyses. Pareto scaling is a common method used in multivariate analysis, particularly in metabolomics. It involves dividing each variable by the square root of its standard deviation, which helps to balance the impacts of variables with high and low variances. PCA was employed first to explore the internal variation within the dataset and identify any potential biomarkers, which indicated the presence of unique features that differentiate these five groups of soy sauce [36].

After the initial PCA analysis, the PLS-DA technique was employed to investigate the separation between the samples further, and identify the variables (features) responsible for the differentiation between the five groups (0-, 2-, 4-, 6- and 8-month fermented soya sauce). The PLS-DA model’s quality enabled us to evaluate the components’ total variance at a confidence level of 95%. A higher total variance signifies a better-quality model for elaborating the differences between the variously aged samples of soya sauce. PLS-DA identified the variables contributing to the differentiation between five groups of soya sauce, which have different maturity times and fermentation effects. Model validation was conducted through CV ANOVA with a significance level of *p* < 0.05. R2X and Q2 demonstrated how well the model fit, and predicted outcomes. This analysis provided valuable insights into the specific features that distinguish among the metabolites present in the soya sauce of different fermentation times, and also aided in further understanding the factors that drive the observed separation [33].

## 3. Results and Discussion

### 3.1. ^1^H-NMR Spectroscopy

**Figure 1:**
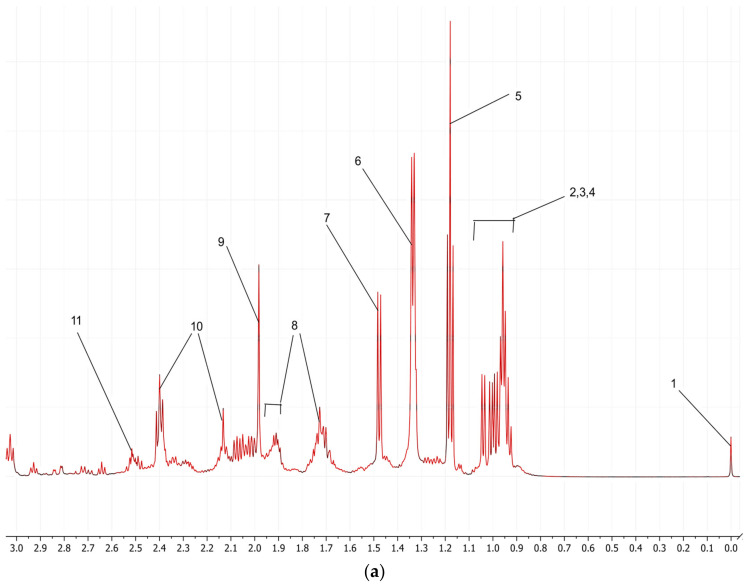

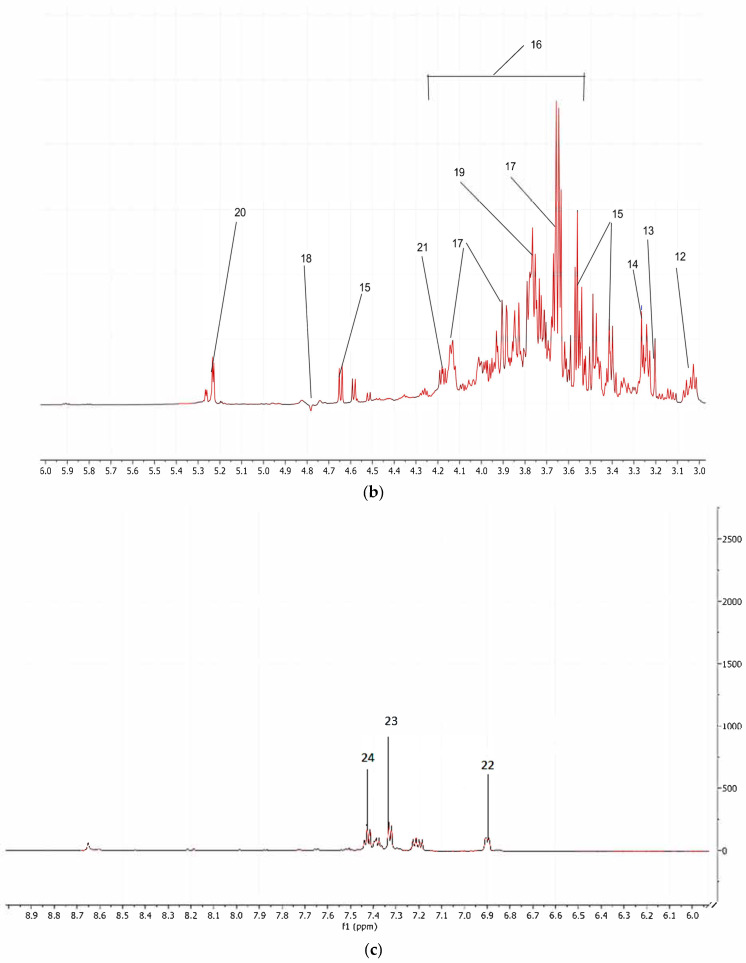
(**a**–**c**): The representative ^1^H-NMR spectra of soya sauce samples. Peaks: 1, TSP; 2, isoleucine; 3, leucine; 4, valine; 5, ethanol; 6, lactate; 7, alanine; 8, lysine; 9, acetate; 10, butyrate; 11, methionine; 12, γ-amino butyric acid; 13, choline; 14, glucose; 15, β-Glucose; 15, fructose; 16, glucose aliphatic region; 17, fructose; 18, water; 19, glycerol; 20, α-Glucose; 21, sucrose; 22, formate; 23, tyrosine; 24, phenylalanine.

After stacking and processing the spectra, the signals in the NMR spectra of soya sauce samples were identified using an in-house database, freely available online software (i.e., HMDB, SDBS), and published data. The identified compounds have been listed in Table 1. A representative spectrum of a soy sauce samples set is shown in Figure 1a–c.

The spectra of five different fermentation times were compared with each other to evaluate the differences among them. The NMR signals of soya sauce of various aging periods show the same pattern. Nonetheless, the differences were observed in the metabolites signals’ compositions and concentrations. The intensities of the signals vary significantly for some compounds as the spectral profiles go from zero months to eight months. The variation in the signal intensity may occur due to the differences in the aging period of soya sauce or the additional flavors and preservatives added during manufacturing. To observe subtle differences and variations in the signal, an expanded view of the 3.0 to 6.0 ppm region is depicted in Figure 1b, since this region mostly covers signals of oligosaccharides and glucose overlapping.

Acknowledging the impacts of sugar signals and their potential interference with detecting and quantifying other compounds in the spectral analysis is crucial. As predominant compounds in complex food items, sugars exhibit signals overlapping with each other and cause ambiguity in the confirmation of compounds present in very low concentrations, like the organic acids that fall in this region. This overlapping effect poses a challenge in accurately quantifying metabolites, as it can introduce variations in the signal intensities among different samples [37].

### 3.2. Multivariate Statistical Analysis

**Figure 2:**
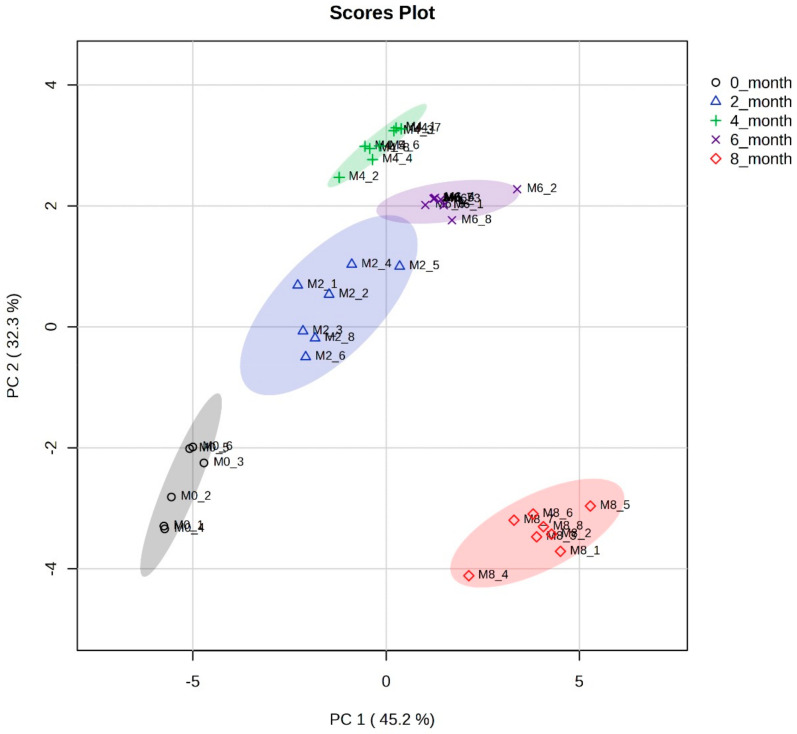
Score scatter plot of PCA showing a clear separation of five groups of soy sauce samples affected by an 8-month fermentation.

To analyze the metabolite differences in soy sauce during fermentation, 37 samples were studied using ^1^H-NMR spectroscopy combined with multivariate statistical analysis. PCA is an unsupervised technique commonly used for analyzing multivariate data, including NMR spectroscopic data. PCA aims to simplify the data by reducing their dimensionality while retaining as much relevant information as possible. It transforms the original variables into a new set of uncorrelated variables called principal components [38]. The score scatter plot of PCA in Figure 2 shows the complete separation of five groups of soy sauce fermented for different periods, indicating differences in the composition of samples at various periods of fermentation. The loading plot of PCA shown presents the metabolites responsible for separating five different sample sets of soy sauce belonging to five different fermentation periods.

The score scatter plots of soy sauce samples with different fermentation times show variables at PC1 and PC2, with 45.2% and 32.3%, respectively. In PC1, clear distinctions can be observed among samples fermented for different periods (0–8 months), identifying five distinct groups. Specifically, samples fermented for 0, 2, and 4 months are positioned on the negative side of PC1, while samples fermented for 6 and 8 months are positioned on the positive side of PC1. Notably, there were significant variations in the PCA values among all five time points.

Regarding PC2, notable differences in levels were observed. Samples fermented for 0 and 8 months are located on the negative side of PC2, whereas samples from months 2, 4, and 6 of fermentation are on the positive side of PC2. Among all the samples, those from the 4th month of fermentation exhibit the highest PC2 value. Overall, the analysis of PC1 and PC2 in this context reveals distinct patterns and variations in the samples based on their fermentation periods.

These findings suggest that the fermentation time plays a significant role in shaping the metabolic characteristics of the samples, as evidenced by their positions and values in the PCA plot. This differentiation indicates the presence of specific metabolic characteristics in five types of samples. Hence, the PCA analysis demonstrates the existence of unique compositional variations between soy sauce samples of various fermentation ages.

The PCA scatter plot demonstrates the most significant differences between the soy sauce samples fermented for eight months and those fermented for shorter periods. PLS-DA models were computed to further explore and utilize the distinct metabolomic characteristics of the different samples based on their fermentation periods.

PLS-DA plots were employed to identify the essential variables responsible for discriminating among the soy sauce samples with different aging times. These plots highlight the influential variables that contribute significantly to the differentiation of the soy sauce samples based on their fermentation durations. By analyzing the PLS-DA loading plots, one can gain insights into the specific metabolites or features that drive the discrimination, and understand their importance in distinguishing between soy sauce samples [39].

**Figure 3:**
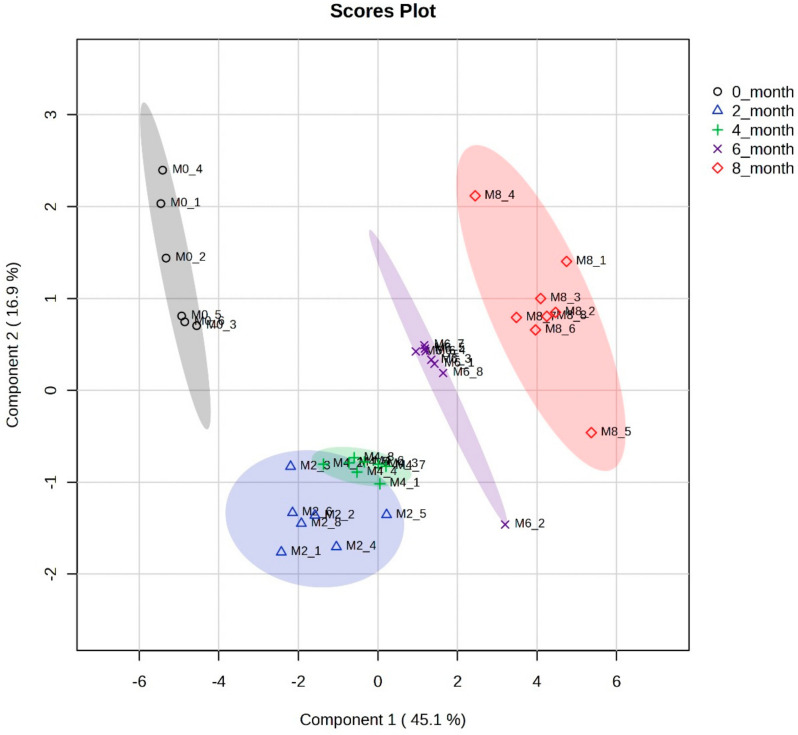
PLS-DA score scatter plot of soy sauce samples affected by different fermentation times.

**Figure 4:**
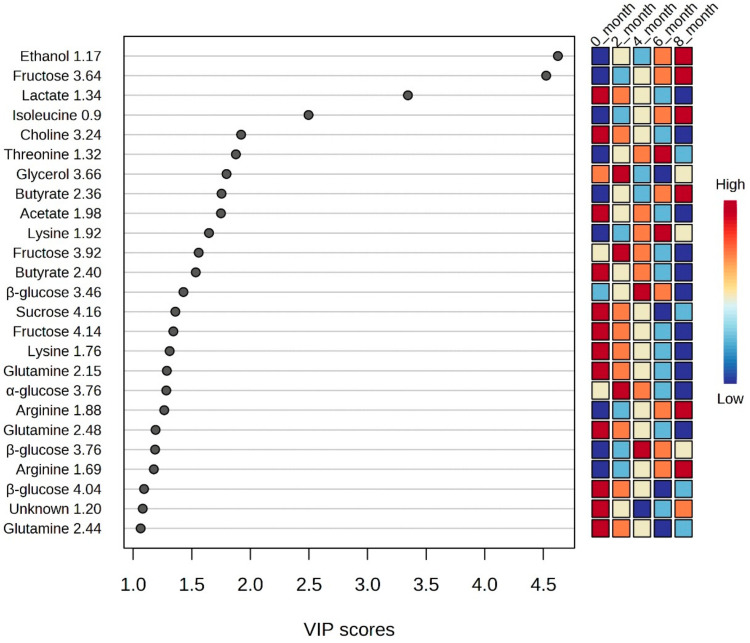
The metabolites exhibiting the highest variation based on the VIP scores from PLS-DA of soy sauce samples having different fermentation times.

PLS-DA models were analyzed to study the metabolic profiles of samples from five different data sets of different fermentation times, further elaborating on the differences among the five data classes. The PLS-DA scatter plot shown in Figure 3 presents a very good separation, with R2X 96% and Q2 93%, which indicates the goodness-of-fit and the overall predictive ability of the data plots. The metabolites exhibiting the highest variation were selected based on loading plots from PLS-DA, as presented in a VIP score plot in Figure 4.

**Figure 5:**
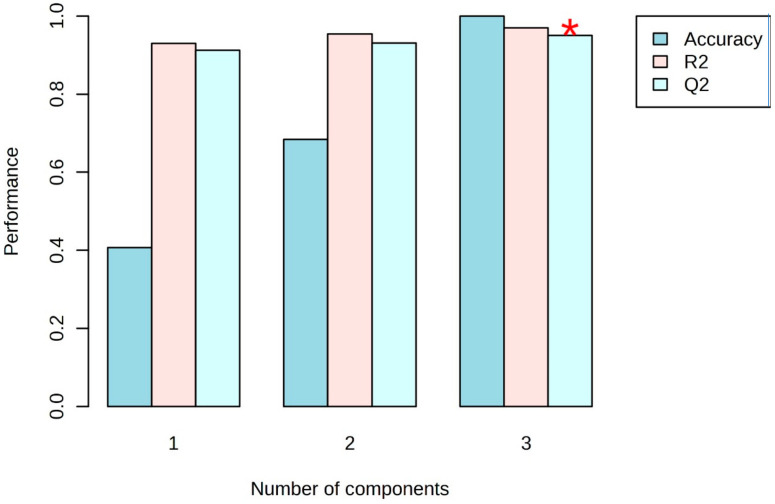
PLS-DA classification using different numbers of components. The red asterisk indicates the best classifier showing, the performance of R2X 96% and Q2 93%.

R2X and Q2 are valuable metrics in evaluating a PLSDA model’s quality and predictive ability, providing insights into how well the model captures the variance in the data and its ability to make accurate predictions. R2X quantifies how well the model captures the overall data variability. The R2X values range from 0 to 1, where a value of 1 indicates that the model explains all the variance in the data. Meanwhile, Q2 assesses how well the model predicts the original data when the model is cross-validated as shown in Figure 5. Cross-validation involves systematically leaving out a portion of the data during model training and evaluating the model’s predictive performance on the excluded data.

**Figure 6:**
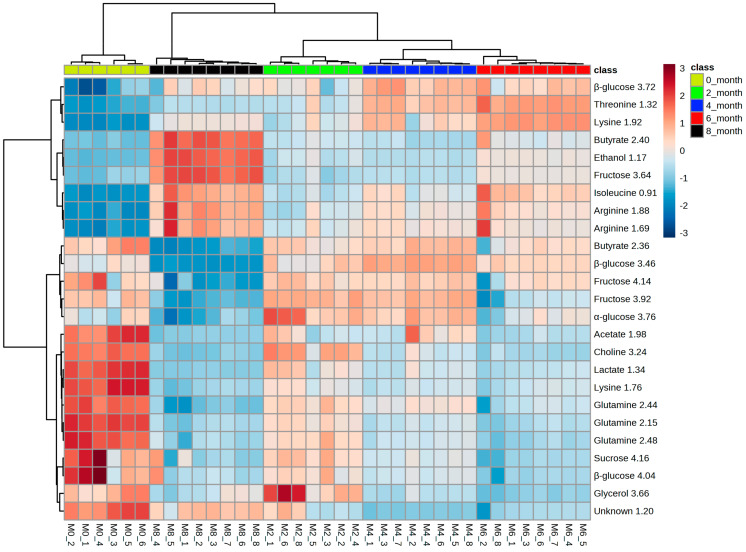
The heat map of the top 25 most significant metabolites of 138 features of the PLS-DA model.

**Figure 7:**
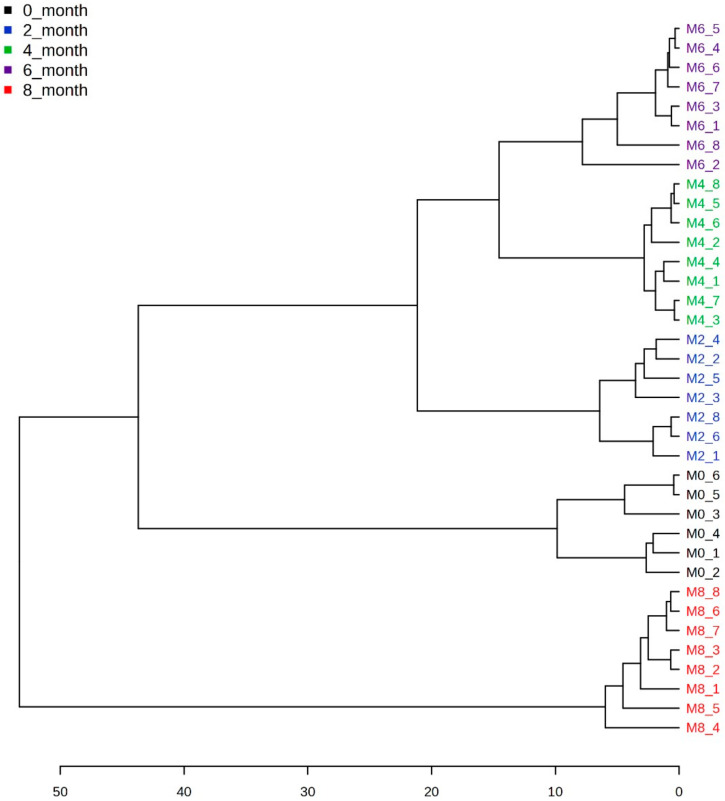
The cluster tree clearly shows that five datasets do not overlap with each other.

The heat map of the top 25 most significant metabolites out of 138 features of the PLS-DA model shown in Figure 6 clarifies the metabolites causing the separation. How the concentrations of these metabolites changed during the different fermentation times and the cluster tree presented in Figure 7 show that the datasets do not overlap. Amino acids like lysine, threonine, isoleucine, choline, alanine, arginine, glutamine, and methionine, along with acetate, glutamate, butyrate, glucose, fructose, and sucrose, were observed to significantly change their concentration in soy sauce samples of different fermentation durations. Ethanol and glycerol were also included in metabolites, which vary considerably during fermentation.

### 3.3. Complex Carbohydrates to Simple Sugars

The trends of the carbohydrates in soy sauce samples of various aging periods were found to be different for various sugars. The three most important sugars, glucose, fructose, and sucrose, were observed in all the samples belonging to different age times of 0 to 8 months. In the starting months, there were increasing levels of *β*-glucose from 0 to 4 months; after the 4th month, as the fermentation passed, the sugar level also dropped, and the lowest level of *β*-glucose was observed in month 8 of fermentation, while the concentration of fructose was found to be increased in a straight-line slope from 0 to 8 months of fermentation.

Earlier research has indicated that a decrease in sugar concentration after some period of fermentation in soy sauce or any fermented product indicates higher carbohydrate consumption by microorganisms during fermentation. This observation aligns with previous studies that demonstrated the increased consumption of oligosaccharides by wine yeast in grape wines over time [29]. Notably, a significant decrease in the levels of oligosaccharides was observed during a 6-month aging period after alcoholic fermentation [40].

In addition to their role in fermentation, carbohydrates in soy sauce play a beneficial role in the production of immunoglobulin A (Ig A) both in vivo and in vitro. *Tetragenococcus halophilus*, a halophilic lactic acid bacterium (LAB), is actively involved in soy sauce fermentation and possesses immunomodulatory activity [41]. Bacteria break down soybean and wheat starches into polysaccharides and oligosaccharides over time. This process enhances the nutritional profile of soy sauce, making it an important dietary source for enhancing host defenses. However, optimizing the fermentation process to retain the appropriate amount of sugars necessary to maintain the distinctive characteristics of pure fermented soy sauce is crucial. Striking the right balance in sugar content is important to preserve soy sauce’s unique properties and flavor profile while maximizing its potential as a dietary source for enhancing immune responses. Higher levels of sugars in the soy sauce support the hypothesis that the fermentation duration of the product was short [40].

The level of sucrose in the soy sauce samples was found to be higher in earlier months, and decreased gradually as the fermentation time went from month 0 to month 8. Sucrose is present in the raw ingredients used to make soy sauce, such as wheat, which contains this disaccharide. However, it is important to note that the sucrose levels in the final product may vary depending on the manufacturing process and the duration of fermentation.

Caramel is commonly used as an additive in soy sauce production to enhance flavor and color. It is typically manufactured by heating a combination of sugars, including maltose, lactose, and sucrose. During the fermentation of caramel, the lactose in the mixture breaks down into its monomers, glucose and galactose [42]. However, the sucrose and maltose remain relatively intact and abundant. Maltose is a disaccharide composed of two glucose molecules, while sucrose is a disaccharide consisting of glucose and fructose. The resulting mixture of caramel contains a combination of sugars, including glucose, fructose, maltose, and sucrose. This caramel mixture is added to soy sauce to provide a richer flavor profile and a dark brown color [43]. Therefore, soy sauce may contain small amounts of sucrose resulting from fermentation; the primary sugars are usually glucose and fructose, which are starch-breakdown products. The sucrose levels in soy sauce are typically relatively low compared to other sugars.

Soy sauce fermented for a longer duration, precisely eight months, exhibited significantly lower levels of both *α*-glucose and *β*-glucose compared to samples fermented for a shorter period. This observation indicates that the production of organic soy sauce involves meticulous and extended fermentation procedures. The decrease in glucose levels suggests that the microorganisms responsible for fermentation efficiently utilized glucose as an energy source during the prolonged fermentation process.

### 3.4. Narrative of Ethanol and Glycerol

*Zygosaccharomyces rouxii*, a yeast strain, plays a crucial role in soy sauce fermentation by producing ethanol and glycerol. The key raw materials for synthesizing these compounds are wheat and soybean starch. Since the starch contents in wheat and soybeans can vary, the amounts of ethanol and glycerol generated during fermentation may differ accordingly [4].

The duration and temperature of fermentation also impact the evaporation of ethanol from the mixture. Consequently, soy sauce samples subjected to more extended fermentation periods contain significantly lower ethanol levels than those fermented for shorter durations. This observation indicates that prolonged fermentation decreases ethanol content in the final soy sauce product [44].

The current study found that the ethanol concentration increased as aging extended from 0 to 8 months. It is important to note that while ethanol is naturally produced during fermentation, commercial soy sauce production often involves intentionally adding edible ethanol as a preservative. This practice helps maintain product stability and prolong shelf life. Thus, the ethanol levels in soy sauce can be influenced by both the natural fermentation process and the deliberate addition of ethanol during manufacturing.

### 3.5. Proteolysis Giving Birth to Amino Acids

Amino acids are primarily derived from raw materials such as soybean and wheat during the meju formation and sauce fermentation processes. Proteolysis occurs during the fermentation of soybeans and wheat in soy sauce production, which breaks down proteins into amino acids. Several amino acids are produced during this process. Initially, microorganisms utilize these amino acids as a nitrogenous source for their growth. As fermentation progresses, more amino acids are also grown due to enzymes breaking down soy or wheat proteins into amino acids [23].

The most abundant amino acids in soy sauce include isoleucine, threonine, lysine, and arginine [45]. Isoleucine and arginine showed a noticeable change in concentration during the fermentation process of 8 months. The concentration of these amino acids increased in a straight-line slope from 0 to the 8 months of fermentation. The levels of threonine and lysine increased as the fermentation matured, and they exhibited the highest concentrations at 6 months, after which a slight fall was seen during the 8th month.

Some other amino acids that showed no significant change in their concentration during the fermentation process were also reported. Still, their very small peaks were observed in all samples with different fermentation times. Glycine, alanine, leucine, methionine, valine, phenylalanine, tyrosine, glutamic acid, and aspartic acid were included, while choline and glutamine showed distinct reductions in their concentration as the fermentation progressed.

These amino acids are essential in shaping the sauce’s distinctive taste and aroma. Glutamic acid is mainly responsible for the umami taste, infusing the sauce with its savory and rich flavor [46]. Aspartic acid also contributes to the overall flavor profile of soy sauce, enhancing its taste complexity. Glycine, known for imparting sweetness, is a flavor enhancer in the sauce. Alanine further adds to the mild sweetness and helps achieve a balanced flavor. Leucine brings a slight bitterness and aromatic essence to the sauce. Valine plays a crucial role in the overall flavor and aroma profile, making soy sauce a culinary delight appreciated worldwide for its unique taste sensation [18].

These amino acids are formed due to microorganisms’ enzymatic breakdown of proteins during fermentation. Still, the specific compositions and proportions of amino acids may vary depending on factors such as fermentation conditions, fermentation duration, and the strains of microorganisms used to produce soy sauce.

Consuming soy sauce provides a source of essential amino acids that are important for various bodily functions, including muscle growth and repair, hormone synthesis, and enzyme production; it thus contributes to overall health, and may help reduce the risk of chronic diseases.

### 3.6. Footsteps of Lactate, Butyrate and Acetate

The butyrate, lactate, and acetate concentrations varied significantly during fermentation from month 0 to month 8. Organic acids are produced during fermentation through the metabolic activities of various microorganisms. Lactic acid bacteria (LAB) convert sugars, such as glucose, into lactic acid through a series of enzymatic reactions, and specific bacteria, such as *Clostridium* species, can ferment carbohydrates and produce butyric acid as a metabolic byproduct [47]. Similarly, acetic acid bacteria oxidize ethanol into acetic acid through acetic acid fermentation.

The current study found a decrease in the concentrations of these salts of organic acids during the fermentation process, identifying lower levels of lactic acid, butyric acid, and acetic acid in the fermented soy sauce samples.

The lower levels of organic acids found during the fermentation period from 0 to 8 months could be due to various factors, of which utilization by microorganisms is the most significant. Microorganisms, such as lactic acid bacteria, may consume lactic acid as a substrate for further metabolism or energy production, decreasing its concentration. Certain organisms, particularly those involved in secondary fermentation, utilize butyric acid and acetic acid as substrates for their growth and metabolic processes. These microorganisms can use these acids as energy sources, or convert them into other compounds. As a result, the concentrations of these acids decrease during fermentation due to their consumption by these microorganisms.

Lactic acid can also be converted into other metabolites, such as acetic or butyric acid, through specific metabolic pathways involving certain bacteria [47]. Similarly, butyric acid and acetic acid can be converted into various compounds through specific metabolic pathways involving certain bacteria. This conversion process may decrease the concentration of butyric acid and acetic acid, while increasing the concentrations of other metabolites, such as acetic acid or gases such as hydrogen and carbon dioxide. Additionally, pH adjustments in the fermentation process, such as adding alkaline substances, can cause these organic acids to react, lowering their concentration.

## 4. Conclusions

The metabolites present in soy sauce during different fermentation durations, and how their concentrations varied with fermentation maturity, were confirmed efficiently using ^1^H-NMR spectroscopy coupled with multivariate statistical analysis. The multivariate analysis of soy sauce samples fermented for different periods revealed distinct patterns and variations. Samples fermented for 0, 2, and 4 months were significantly different from those fermented for 6 and 8 months. The key metabolites that exhibited variations included amino acids (lysine, threonine, isoleucine), acetate, glucose, fructose, sucrose, ethanol, glycerol, and lactate. These findings highlight the significant influences of fermentation time over the metabolic characteristics of the samples. They indicate the presence of unique compositional variations among samples with different fermentation periods. They will help us to optimize the fermentation duration in order to achieve the best taste, aroma, and quality of soy sauce. Thus, the results will help us in optimizing each product’s taste, flavor, smell, and nutritional value. Hence, it is necessary to comprehend the chemical composition of soya sauce at different fermentation stages in order to interpret its nutritional and technological properties, and to optimize the conditions required for the best production of traditional Chinese soya sauce. The preliminary findings of the present study can lead to further detailed studies of soy sauce fermentation over prolonged periods of aging, sometimes reaching up to years.

## Figures and Tables

**Table 1:** Assignment of metabolites based on ^1^H-NMR spectroscopy.

Sr. No.	Compound	^1^H-NMR Chemical Shift
1 2 3 4	TSP Isoleucine Leucine Valine	0.00 (s) 0.94 (t) 0.98 (d) 0.99 (d)
5	Ethanol	1.17 (t)
6 7	Lactate Alanine	1.34 (d) 1. 48 (d)
8	Lysine	1.73 (m), 1.91 (m)
9	Acetate	1.98 (s)
10	Butyrate	2.13 (m), 2.39 (m)
11 12	Methionine γ- amino butyric acid	2.50 (t) 3.02 (m)
13 14	Choline Glucose	3.20 (s) 3.25 (t)
15 16	β-Glucose Glucose, aliphatic region	3.40 (t), 3.59 (d), 4.64 (d) 3.50–4.25
17 18	Fructose Water (solvent)	3.64 (m), 3.90 (d), 4.14 (m) 4.78 (s)
19	Glycerol	3.76 (m)
20	α-Glucose	5.22 (d)
21 22 23 24	Sucrose Formate Tyrosine Phenylalanine	4.16 (d) 6.88 (s) 7.30 (d) 7.42 (m)

s = singlet, d = doublet, t = triplet, m = multiplet.

## Data Availability

No new data were created or analyzed in this study. Data sharing is not applicable to this article.
